# Wuguchong Bioactive Peptides Promote Wound Healing via Hedgehog Pathway‑Mediated Fibroblast Modulation

**DOI:** 10.1002/cbdv.71671

**Published:** 2026-10-21

**Authors:** Kun Li, Yunpu Sun, Lijie Pang, Tiantian Gao, Yunpeng Diao, Shuyuan Fan

**Affiliations:** ^1^ College of Chemistry and Chemical Engineering Liaoning Normal University Dalian China; ^2^ College of Pharmacy Dalian Medical University Dalian China; ^3^ Dalian Medical University Dalian China

**Keywords:** fibroblasts, hedgehog pathway, wound healing, Wuguchong

## Abstract

The skin undertakes multiple critical physiological functions, including barrier protection and thermoregulation. However, chronic nonhealing ulcers have emerged as a major clinical challenge. In wound repair, skin healing is a highly coordinated, time‐sequential process wherein fibroblasts—key dermal cells—play irreplaceable roles in proliferation, migration, differentiation, and extracellular matrix (ECM) synthesis. When external environmental factors induce fibroblast dysfunction, this can lead to impaired epithelialization, thereby resulting in persistent wound nonclosure, which may represent a key mechanism underlying the pathogenesis of this condition. Yet current clinical treatments (debridement, anti‐infection, negative pressure drainage, and growth factors) fail to resolve this, with high recurrence and poorly understood molecular mechanisms. To this end, we identified in this study a low‑molecular‑weight bioactive peptide (< 10 kDa) originating from Wuguchong (JY), which is capable of specifically regulating fibroblasts in the wound microenvironment. By modulating the Hedgehog (Hh) signaling pathway, this peptide restores fibroblast function, thereby promoting wound healing and effectively ameliorating the challenge of impaired wound repair. This study not only offers promise for the development of new drugs for treating ulcers, but also provides novel insights and strategies for cell‑based therapies targeting other types of wound healing.

## Graphical abstract

The skin undertakes multiple critical physiological functions, including barrier protection and thermoregulation. However, chronic non‐healing ulcers have emerged as a major clinical challenge. In wound repair, skin healing is a highly coordinated, time‐sequential process wherein fibroblasts—key dermal cells—play irreplaceable roles in proliferation, migration, differentiation, and extracellular matrix (ECM) synthesis. When external environmental factors induce fibroblast dysfunction, this can lead to impaired epithelialization, thereby resulting in persistent wound non‐closure, which may represent a key mechanism underlying the pathogenesis of this condition. Yet current clinical treatments (debridement, anti‐infection, negative pressure drainage, growth factors) fail to resolve this, with high recurrence and poorly understood molecular mechanisms. To this end, we identified in this study a low‑molecular‑weight bioactive peptide (<10 kDa) originating from Wuguchong (JY), which is capable of specifically regulating fibroblasts in the wound microenvironment. By modulating the Hedgehog signaling pathway, this peptide restores fibroblast function, thereby promoting wound healing and effectively ameliorating the challenge of impaired wound repair. This study not only offers promise for the development of new drugs for treating ulcers but also provides novel insights and strategies for cell‑based therapies targeting other types of wound healing.

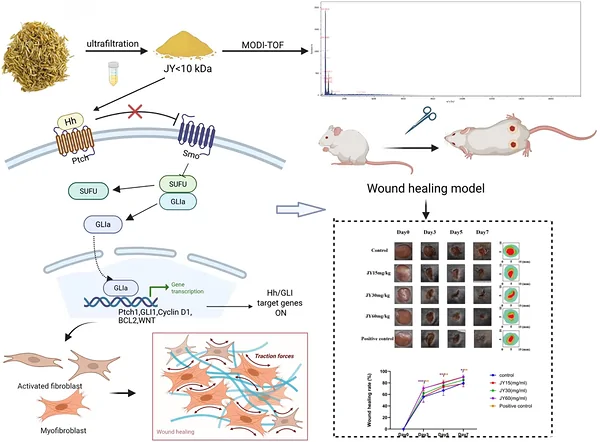

## 1 Introduction

Skin wound healing is a coordinated biological process involving hemostasis, inflammation, proliferation, and tissue remodeling [1, 2]. Successful repair requires the timely interaction of multiple cell types and signaling pathways to restore tissue continuity and function [3]. Fibroblasts are particularly important during the proliferative and remodeling phases because they migrate into the injured area, proliferate, produce extracellular matrix (ECM), and contribute to granulation tissue formation [4, 5]. In response to local signals, a subset of fibroblasts differentiates into contractile myofibroblasts, which express α‐smooth muscle actin (α‐SMA), generate mechanical tension, and promote wound contraction [6, 7, 8, 9, 10, 11]. Therefore, appropriate regulation of fibroblast proliferation, migration, activation, and matrix synthesis is essential for efficient skin repair.

Although acute cutaneous wounds generally heal through an orderly and self‐limited process, delayed or impaired repair may occur when cellular responses are insufficient, dysregulated, or exposed to unfavorable local conditions [12, 13]. Current wound‐management strategies, including debridement, infection control, negative‐pressure therapy, biological dressings, and growth‐factor‐based interventions, can improve clinical outcomes, but their effectiveness varies according to wound type and patient condition. Identifying natural products that can enhance the activity of key repair cells may therefore provide useful leads for developing new wound‐healing interventions [14, 15, 16, 17].

The Hedgehog (Hh) signaling pathway is an important regulator of tissue development, homeostasis, and repair [18, 19]. Mammals express three Hh ligands: Sonic hedgehog (Shh), Indian hedgehog (Ihh), and Desert hedgehog (Dhh), which have distinct biological functions. In the skin, Shh signaling participates in hair follicle cycling, sebaceous gland development, and tissue regeneration [20, 21]. Ihh is mainly associated with chondrocyte proliferation and endochondral ossification, whereas Dhh contributes to testicular development and peripheral nerve organization [22, 23]. In the canonical Hh pathway, ligand binding relieves Patched1 (PTCH1)‐mediated inhibition of Smoothened (SMO), leading to the activation of GLI transcription factors and the expression of downstream genes involved in cell proliferation, survival, and tissue remodeling [24, 25]. Increasing evidence indicates that Hh signaling is activated after skin injury and contributes to dermal fibroblast proliferation, migration, and skin appendage regeneration [21]. These findings suggest that modulation of Hh signaling may influence fibroblast behavior during cutaneous wound repair.

Bioactive substances (such as bioactive peptides [26, 27, 28]) derived from natural sources have attracted considerable interest as potential wound‐healing agents because of their structural diversity and biological activity [29, 30, 31]. Wuguchong (JY), derived from the desiccated larvae of *Chrysomya megacephala* and related species in the family Calliphoridae, has traditionally been used in wound management. Previous studies have mainly focused on the debridement‐related effects of live maggots or the antimicrobial and other biological activities of crude maggot‐derived preparations [32, 33]. However, there remains a paucity of evidence regarding the components of JY with potential tissue‑repairing activity, as well as their underlying mechanisms.

In the present study, we prepared a JY fraction with a molecular weight below 10 kDa, referred to as JY < 10 kDa, and evaluated its effects on cutaneous wound repair. Using a mouse acute full‐thickness excisional wound model and L929 fibroblast‐based assays, we examined wound closure, tissue regeneration, fibroblast proliferation and migration, fibroblast activation, and collagen production. We further investigated the involvement of the Hh pathway by analyzing PTCH1, GLI1, and Cyclin D1 expression and by using the SMO inhibitor vismodegib. This study aims to clarify the potential role of JY < 10 kDa in acute skin wound healing and to determine whether Hh pathway‐associated regulation of fibroblast function contributes to its biological activity.

## 2 Materials and Methods

### 2.1 Experimental Animals

Twenty SPF‐grade male C57BL/6 mice (22–24 g) were obtained from Cyagen Biosciences (Gu'an, China) and housed under standard conditions at the Experimental Animal Center of Dalian Medical University, with ad libitum access to food and water. All procedures complied with the Regulations for the Administration of Laboratory Animals issued by the State Science and Technology Commission of China.

### 2.2 Experimental Cells and Treatment

The mouse fibroblast L929 cell line (CVCL_0462, iCell‐m026) was obtained from Shanghai Saibai Kang Biotechnology Co. Ltd. (China) and cultured in Eagle's Minimum Essential Medium (MEM) containing 10% fetal bovine serum (FBS) and 1% penicillin–streptomycin. L929 were treated with JY solution and Hh pathway inhibitor vismodegib (10 µM) for 24 h [34].

### 2.3 Preparation of JY < 10 KDa Bioactive Component

Wuguchong (JY) is derived from the desiccated larvae of *C. megacephala* and other allied species within the family Calliphoridae. The dried JY insect used in this study was sourced from Leshan, Sichuan Province, China. The production license (Liao 20150128) was issued by Dalian Medical University School of Pharmacy, and it was identified and provided by them (product number: 241101). Dried JY (100 g) was weighed, ground, and extracted by refluxing with 300 mL triple‐distilled water at 70 °C three times (1 h each). The combined supernatants were concentrated by rotary evaporation and lyophilized. The crude extract was redissolved in water (< 100 mg/mL), filtered through membranes (0.22 µm), and ultrafiltered using a 10 kDa centrifugal filter (4500 × g, 40 min). The filtrate (JY<10 kDa) and retentate (JY > 10 kDa) were collected separately and lyophilized for subsequent use. The 10 kDa as a bioactive fraction containing small molecules (primarily peptides).

For in vitro assays, the total aqueous extract of JY and its JY > 10 kDa and JY < 10 kDa fractions were dissolved in dimethyl sulfoxide (DMSO) as 256 mg/mL stock solutions, the final concentration of DMSO was 0.1% (v/v), and their protein contents were determined using the bicinchoninic acid (BCA) assay. For in vivo experiments, JY powder was dissolved in a glycerol–water vehicle (1:1, v/v) to concentrations of 15, 30, and 60 mg/mL. Each wound was treated with 0.1 mL of formulation containing JY solution at concentrations corresponding to final doses of 15, 30, and 60 mg/kg. Kangfuxin Liquid (Tianshuxin), a commercial preparation of ethanol extract from the dried body of *Periplaneta americana L*., was purchased from Hunan Kelun Pharmaceutical Co., Ltd. (Yueyang, Hunan, China; approval No. Z43020995; Lot No. M240317) and used as the positive control as received, and each wound was treated with 0.1 mL. All formulations were prepared fresh before use, vortexed, and applied topically to wound sites.

### 2.4 Animal Grouping, Model Establishment, and Treatment

All animal procedures were approved by the Animal Ethics Committee of Dalian Medical University (Approval No. XL250610065). Mice were anesthetized, and after loss of the pain reflex was confirmed, the dorsal hair was shaved. A full‐thickness excisional wound (1 cm in diameter) was created on the dorsal skin using sterile surgical scissors. The mice were randomly divided into five groups (*n* = 4): vehicle control (glycerol–water, 1:1, v/v), low‑dose (15 mg/kg), medium‑dose (30 mg/kg), high‑dose (60 mg/kg) JY < 10 kDa, and a positive control (Kangfuxin Liquid). Each wound received a daily topical application of 200 µL of the corresponding formulation for 7 consecutive days. Wound healing was monitored by photography on days 0, 3, 5, and 7. Wound areas were measured using ImageJ, and the healing rate was calculated as follows:

$$\begin{array}{ccc} \mathrm{Healing}\mathrm{rate}\left(\%\right) & = & \left[\left(\text{Initial area}\;-\;\text{Area on day}\;n\right)/\;\text{Initial area}\right]\\ & & \times \;100\%. \end{array}$$

Statistical analysis was performed using one‑way ANOVA. On day 7, all mice were euthanized, and skin tissue from the wound area was collected for subsequent histological and molecular analysis.

### 2.5 Histological and Immunological Analyses

#### 2.5.1 Hematoxylin–Eosin (H&E) Staining and Masson Staining

Skin tissue samples harvested on day 7 were fixed in 4% paraformaldehyde for 24 h, dehydrated through a graded ethanol series, cleared in xylene, and embedded in paraffin [35]. Sections (5–8 µm) were prepared for staining. For hematoxylin and eosin (H&E) staining, sections were deparaffinized, rehydrated, stained with hematoxylin (5 min), differentiated in 1% acid ethanol (30 s), blued in running water (10 min), and counterstained with eosin (1.5–3 min). After dehydration and clearing, sections were mounted. For Masson's trichrome staining, sections were baked at 65°C for 2 h, deparaffinized, and rehydrated. Staining was performed sequentially with 2.5% potassium dichromate, hematoxylin, ponceau acid fuchsin, 1% phosphomolybdic acid (differentiation), and aniline blue. Sections were then dehydrated, cleared, and mounted. All stained sections were examined under a light microscope to evaluate inflammatory cell infiltration, epithelial regeneration, and collagen deposition.

#### 2.5.2 Immunohistochemistry and Immunofluorescence Staining

For immunohistochemistry (IHC), deparaffinized and rehydrated tissue sections underwent antigen retrieval and were blocked with 5% BSA for 30 min, followed by incubation with primary antibodies (α‐SMA and Collagen I) at 4°C overnight and then with HRP‐conjugated secondary antibodies for 1 h at room temperature. For immunofluorescence (IF) staining of L929 cells, after fixation with 4% paraformaldehyde and permeabilization with 0.1% Triton X‐100, cells were blocked and incubated with anti‐PCNA primary antibody at 4°C overnight, followed by appropriate fluorescent secondary antibodies for 1 h at room temperature. In both procedures, nuclei were counterstained with DAPI, mounted with anti‐fade medium, and visualized under a fluorescence microscope.

### 2.6 Cell Function Assays

#### 2.6.1 EdU Proliferation Assay

L929 cells were seeded in 96‐well plates at 1 × 10^5^ cells/mL, adhered for 24 h, and then serum‐starved in fresh serum‐free medium for an additional 24 h. After starvation, cells were treated with various concentrations of JY < 10 kDa for 24 h. Cell proliferation was assessed using an EdU assay kit, where cells were incubated with EdU, fixed, and stained with a fluorescent azide conjugate. Nuclei were counterstained with Hoechst 33342. Images were acquired by fluorescence microscopy, and the percentage of EdU‐positive cells was quantified to determine the proliferation rate.

#### 2.6.2 Scratch Wound Healing Assay and Transwell Migration Assay

For the scratch wound healing assay, L929 cells were seeded in six‐well plates and cultured to approximately 80% confluence. A uniform scratch was made with a sterile 200 µL pipette tip. After washing with PBS, cells were treated with various concentrations of JY < 10 kDa. Scratch images were taken at 0 and 24 h, and the migration rate was quantified by measuring the reduction in scratch area. For the transwell migration assay, L929 cells in serum‐free medium were seeded into the upper chamber of a 24‐well transwell plate (8 µm pores), with the lower chamber containing medium with 20% FBS as a chemoattractant. After 24 h, nonmigrated cells were removed, and cells on the lower surface were fixed with 4% paraformaldehyde, stained with 0.1% crystal violet, visualized under an inverted microscope, and counted in five random fields per well.

### 2.7 Western Blot Analysis

Tissue homogenates or cultured cells were lysed with RIPA buffer containing protease inhibitors, and total protein was extracted by centrifugation. Protein concentration was determined using a BCA assay. Equal amounts of protein (20–30 µg per lane) were separated by SDS‐PAGE and transferred onto PVDF membranes. After blocking with 5% non‐fat milk in TBST for 2 h at room temperature, membranes were incubated overnight at 4°C with primary antibodies against PTCH1, GLI1, Cyclin D1, α‐SMA, Collagen I, PCNA, and β‐Actin (loading control). Following washes with TBST, membranes were incubated with HRP‐conjugated secondary antibodies for 1.5 h at room temperature. Protein bands were visualized using an ECL reagent, and band intensities were quantified by densitometric analysis with ImageJ software.

### 2.8 Statistical Analysis

All quantitative data are expressed as mean ± SD from at least three independent experiments. Statistical analyses were performed using GraphPad Prism 8.0. Differences among multiple groups were analyzed by one‐way ANOVA followed by a suitable post‐hoc test, while comparisons between two groups were conducted using an unpaired, two‐tailed Student's *t*‐test. A *p* value < 0.05 was considered statistically significant.

## 3 Results and Discussion

### 3.1 JY < 10 kDa Component Promotes Fibroblast Viability and is Identified as the Core Active Fraction

To identify the bioactive fraction of JY, the aqueous extract was separated by ultrafiltration into fractions with molecular weights below and above 10 kDa. The JY<10 kDa fraction was subsequently characterized by MALDI‐TOF mass spectrometry. The detected molecular‐weight distribution ranged from 113.65 to 9108 Da, with prominent signals at 3196.04, 6583, and 7195 Da (Figures 1A and S1). These data confirm that JY < 10 kDa is a heterogeneous low‐molecular‐weight fraction rather than a single purified compound. Accordingly, the term “JY < 10 kDa fraction” is used throughout this manuscript rather than implying that the material represents one defined small molecule or a single peptide.

**FIGURE 1:**
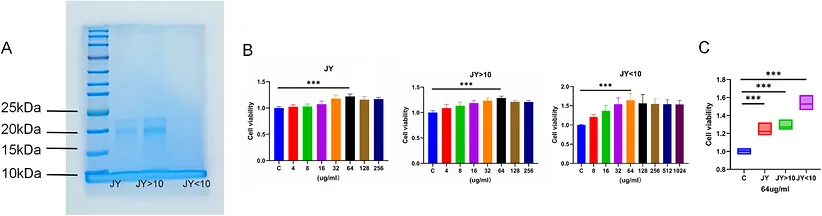
Extraction and pharmacological efficacy evaluation of small‐molecule bioactive compounds from JY. (A) Results of the coomassie brilliant blue assay following ultrafiltration extraction. (B) Results of the CCK‐8 cell viability assay on L929 fibroblast cells treated with JY Bioactive components at different concentrations, *n* = 3. (C) CCK‐8 Cell Viability of L929 fibroblasts following treatment with 64 µg/mL JY bioactive components (*n* = 3, *p < 0.05, **p < 0.01, ***p < 0.001).

The effects of the JY fractions on L929 fibroblasts were evaluated using the CCK‐8 assay. JY<10 kDa increased fibroblast viability in a concentration‐dependent manner, with the most pronounced effect observed at 64 µg/mL and no apparent cytotoxicity under the tested conditions (Figure 1B,C). These findings identify JY<10 kDa as the principal fibroblast‐active fraction of the crude extract. The relatively low molecular‐weight distribution may be advantageous for tissue penetration and formulation development; however, the specific constituents responsible for the observed activity remain to be determined. Further chromatographic separation, high‐resolution mass spectrometry, peptide sequencing where appropriate, and nuclear magnetic resonance analysis will be required to define the active chemical entities and their structure–activity relationships.

The identification of a fibroblast‐active fraction provides a mechanistic extension of the traditional use of JY in wound management. Historically, the beneficial effects of medicinal maggots have been associated mainly with biological debridement, in which live larvae remove necrotic tissue and secrete proteolytic enzymes [36]. Increasing evidence indicates that maggot‐derived preparations may also exert antimicrobial, anti‐inflammatory, angiogenic, and tissue‐reparative effects [32, 33]. The present results suggest that the low‐molecular‐weight fraction contributes directly to wound repair by acting on fibroblasts, which are essential effector cells during granulation tissue formation and ECM remodeling.

### 3.2 JY < 10 kDa Accelerates Wound Closure and Improves Tissue Regeneration In Vivo

The wound‐healing activity of JY < 10 kDa was then examined in a mouse full‐thickness excisional wound model. Topical treatment accelerated wound closure in a dose‐dependent manner compared with the vehicle control (Figure 2A,B). By day 7, the high‐dose JY < 10 kDa group exhibited a healing rate comparable to that of the positive‐control group treated with Kangfuxin liquid, and both groups showed a statistically significant reduction in wound area relative to the model control (*p* < 0.05). These findings demonstrate that the JY < 10 kDa fraction retains substantial reparative activity in vivo and that its effect is not restricted to cultured fibroblasts.

**FIGURE 2:**
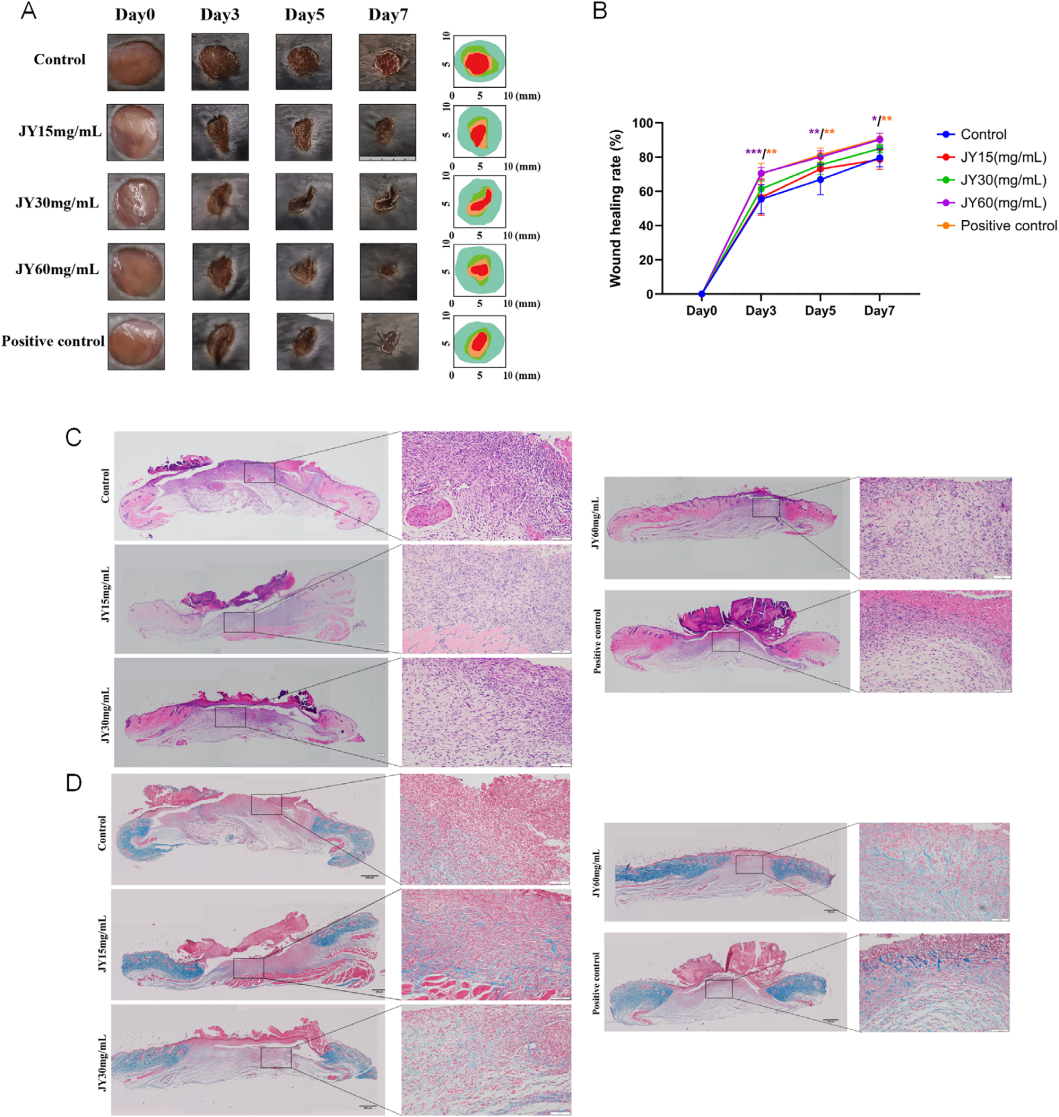
JY < 10 KDa promotes wound healing process recording. (A) Representative images of mouse wound healing over time after treatment with a glycerol–water solution containing JY < 10 kDa components. (B) Wound closure is presented as a percentage of the initial wound area, *n* = 4. *p < 0.05, **p < 0.01, ***p < 0.001 versus the model group for the JY < 10 kDa treatment group versus the model group.

Histological examination provided further evidence of improved tissue repair. Compared with the vehicle‐treated wounds, JY < 10 kDa‐treated wounds displayed more advanced re‐epithelialization, greater epithelial thickness, and more prominent granulation tissue formation, together with reduced inflammatory‐cell infiltration (Figures 2C and S2). Masson's trichrome staining additionally showed increased deposition and improved organization of collagen fibers in the treatment groups (Figures 2D and S3). These histological changes are consistent with accelerated progression through the proliferative phase of healing, during which fibroblast recruitment, granulation tissue formation, and ECM deposition are central events.

The reduction in inflammatory‐cell infiltration is also notable. Although the present study did not directly characterize inflammatory‐cell subsets or inflammatory mediators, excessive or persistent inflammation is a recognized barrier to effective wound closure [37]. The histological findings therefore raise the possibility that JY < 10 kDa may indirectly modulate the wound microenvironment while directly enhancing fibroblast function. This possibility should be investigated in future studies by examining macrophage polarization, inflammatory cytokines, neutrophil persistence, and the resolution phase of inflammation. As the current model simulates acute wounds in healthy mice, the observed outcomes should not be construed as evidence of therapeutic efficacy against chronic ulcers, given that chronic ulcers are characterized by hyperglycemia, hypoxia, vascular impairment, sustained inflammation, and bacterial biofilm, all of which may differentially affect the healing process.

### 3.3 JY < 10 kDa Promotes Wound Healing by Enhancing Fibroblast Activation and Collagen Production

Fibroblasts are recruited to injured tissue, where they proliferate, migrate, produce ECM, and differentiate into myofibroblasts [6]. These processes support granulation tissue formation, wound contraction, and restoration of dermal structure [7, 8, 9, 10]. To elucidate the mechanism through which the JY < 10 kDa fraction modulates fibroblast activity to facilitate wound healing, the expression levels of α‑smooth muscle actin (α‑SMA), type I collagen, and proliferating cell nuclear antigen (PCNA) were assessed in the wound tissues [10, 11].

Immunohistochemical analysis showed weak α‐SMA and type I collagen staining in the vehicle‐control group. In contrast, JY < 10 kDa treatment increased both the density and staining intensity of α‐SMA‐positive cells and enhanced the expression and distribution of type I collagen within the granulation tissue in a dose‐dependent manner (Figures 3A,B, S4, and S5). Western blotting produced consistent results, demonstrating increased levels of PCNA, α‐SMA, and type I collagen in JY<10 kDa‐treated wounds (Figure 3C,D). The relative α‐SMA expression in the high‐dose group reached 3.68 ± 0.25 and was significantly higher than that in the control group (**p < 0.001).

**FIGURE 3:**
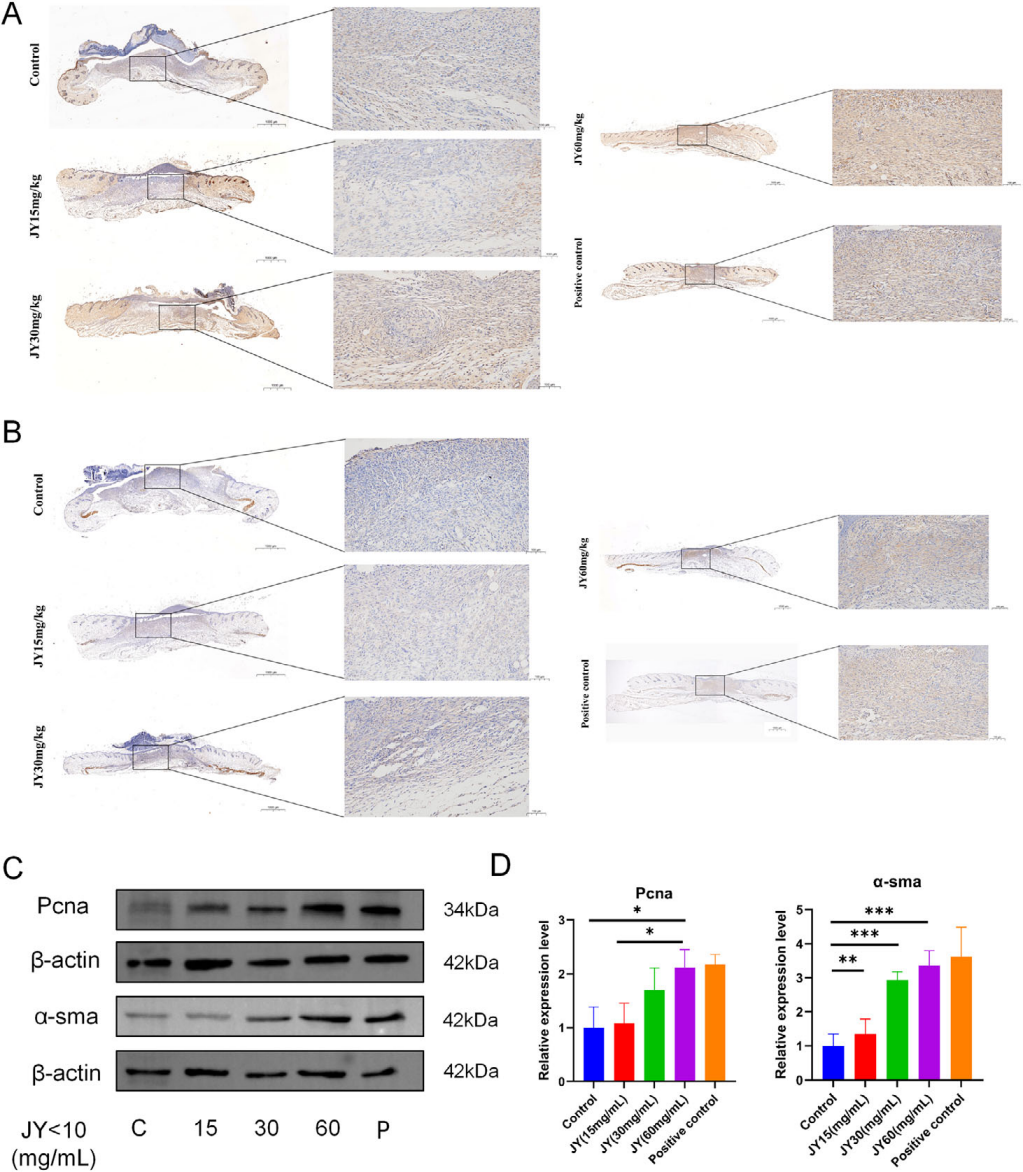
JY < 10 kDa promotes wound healing by facilitating cell activation and collagen production. (A) Immunohistochemical (IHC) analysis of and Collagen I expression was performed on wound tissues treated with JY < 10 kDa, *n* = 3, (Scale bar: 1000 and100 µm). (B) IHC analysis of α‐SMA expression was performed on wound tissues treated with JY < 10 kDa, *n* = 3, (Scale bar:1000 and 100 µm). (C) Expression of cell proliferation marker proteins and collagen proteins in full‐thickness skin wound tissues of mice treated with different concentrations of JY < 10 kDa was analyzed by Western blot. (D) The optical density of all immunoreactive bands was quantified and normalized. Data are from four independent experiments, *n* = 4, *p < 0.05, **p < 0.01, ***p < 0.001.

These findings indicate that JY < 10 kDa promotes the expansion and activation of fibroblastic cells in the wound bed and enhances matrix deposition. PCNA upregulation is consistent with increased proliferative activity, whereas α‐SMA induction suggests an increase in the myofibroblast‐like phenotype. Type I collagen is a major structural component of the repaired dermis and is required for the development of tensile strength. Nevertheless, α‐SMA expression should be interpreted as evidence of myofibroblast activation rather than as a direct measurement of complete fibroblast differentiation or contractile function. Likewise, increased collagen accumulation may be beneficial during the proliferative phase but could contribute to excessive scarring if it persists during remodeling. Longitudinal analysis of collagen turnover, scar architecture, and wound tensile strength will therefore be important for assessing the quality of repair.

### 3.4 JY < 10 kDa Stimulates Fibroblast Proliferation, Migration, and Activation In Vitro

To determine whether the tissue‐level effects were attributable to a direct action on fibroblasts, the effects of JY < 10 kDa were evaluated in L929 cells. EdU incorporation and PCNA immunofluorescence demonstrated that JY < 10 kDa significantly increased fibroblast proliferative activity (Figure 4A). This result is consistent with the increased PCNA expression observed in treated wound tissue and supports the interpretation that JY < 10 kDa enhances the fibroblast population available for tissue repair.

**FIGURE 4:**
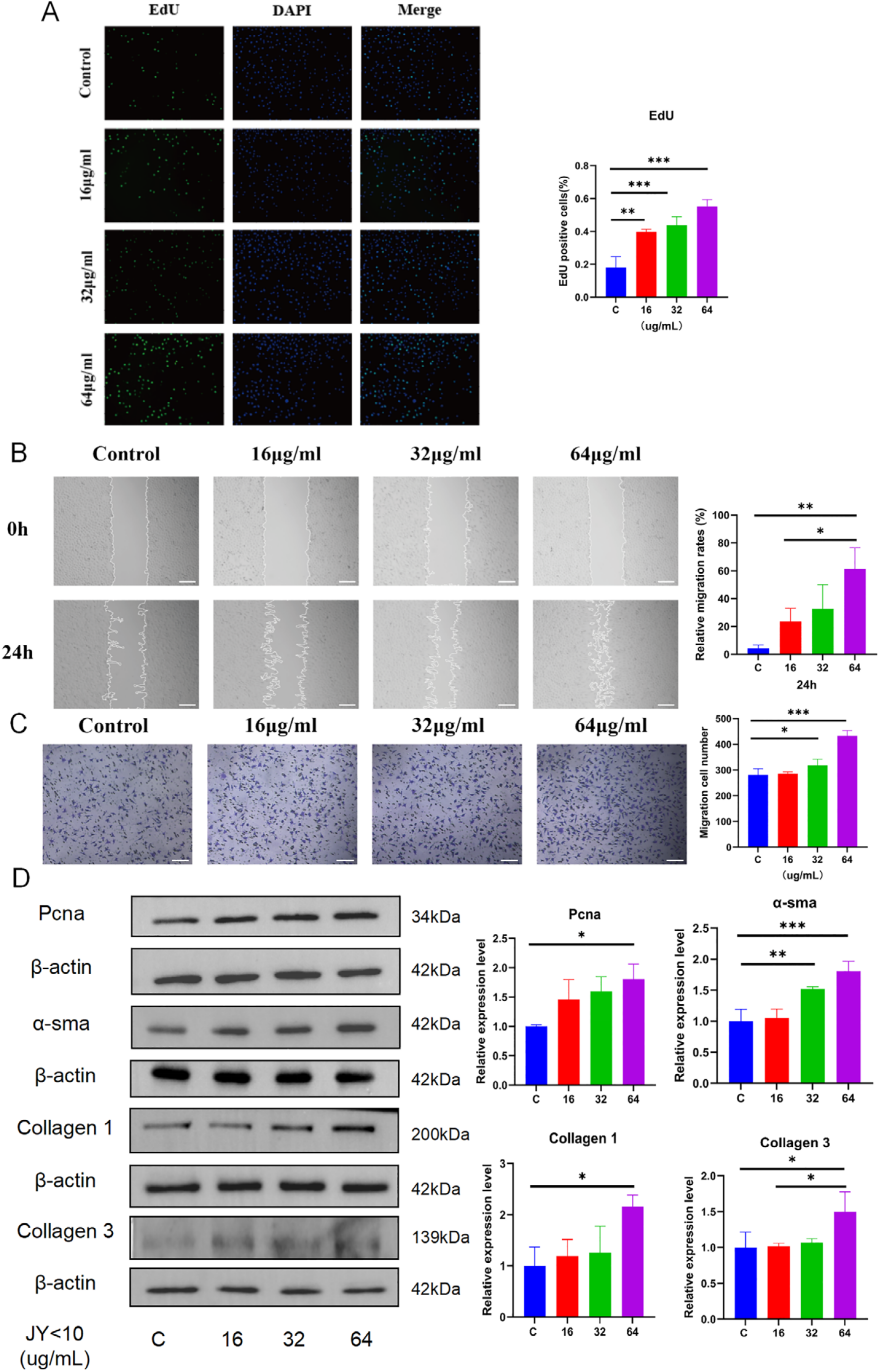
JY < 10 KDa promote wound healing by activating fibroblasts. (A) Quantitative analysis of EdU incorporation revealed that JY < 10 KDa significantly promoted the proliferation of L929 cells. Data are presented as the mean ± standard deviation (SD) from six independent experiments, *p < 0.05, **p < 0.01, ***p < 0.001, (Scale bar: 100 µm). (B) A wound healing (scratch) assay was performed in L929 cells treated with JY < 10 KDa. Representative micrographs of the wound edge at 0 and 24 h post‐treatment with different concentrations of JY < 10 KDa are shown. The migration rate is presented as the percentage of initial wound closure *n* = 3. (C) Cell migration to the lower chamber was assessed using a transwell assay following treatment with different concentrations of JY < 10 KDa. Representative images (Scale bar: 100 µm) and the corresponding quantitative analysis are presented. Data are expressed as the Mean ± SD. *n* = 5, *p < 0.05, **p < 0.01, ***p < 0.001. (D) Expression of proliferation and activation marker proteins in L929 fibroblasts treated with various concentrations of JY < 10 kDa was analyzed by Western blot. The normalized optical density analysis of all immunoreactive bands is presented, quantified from four independent experiments (*n* = 4).*p < 0.05, **p < 0.01, ***p < 0.001.

The effect of JY < 10 kDa on fibroblast migration was assessed using complementary scratch and transwell assays. In the scratch assay, JY < 10 kDa accelerated the reduction of the cell‐free area over 24 h, indicating enhanced lateral migration and/or repopulation of the injured region (Figure 4B). The transwell assay similarly showed an increased number of cells migrating through the porous membrane toward the serum‐containing lower chamber (Figure 4C). The agreement between these two assays strengthens the conclusion that JY < 10 kDa promotes fibroblast motility.

Western blotting further revealed concentration‐dependent increases in PCNA, α‐SMA, type I collagen, and type III collagen in JY < 10 kDa‐treated L929 cells (Figure 4D). Type III collagen is especially relevant during early granulation tissue formation, whereas type I collagen becomes increasingly important during matrix maturation and restoration of tissue strength. Thus, the coordinated induction of α‐SMA and collagen proteins suggests that JY < 10 kDa enhances several complementary fibroblast functions rather than merely increasing cell number. Together, the in vitro findings provide a cellular explanation for the enhanced granulation tissue formation and collagen deposition observed in vivo.

Fibroblasts are heterogeneous and can display distinct phenotypes according to their anatomical origin, developmental lineage, and local wound signals. The L929 cell line provides a useful model for evaluating general fibroblast responses but does not reproduce the full cellular heterogeneity of human skin. Future studies using primary dermal fibroblasts, papillary and reticular fibroblast subpopulations, and human wound‐derived cells will be needed to determine whether JY < 10 kDa preferentially targets a specific fibroblast state.

### 3.5 JY < 10 kDa Activates the Hh Signaling Pathway

The Hh pathway is involved in tissue development, homeostasis, fibroblast behavior, and skin repair [24, 25]. To investigate whether Hh signaling contributes to the activity of JY < 10 kDa, the levels of PTCH1, GLI1, and Cyclin D1 were examined in wound tissue and L929 fibroblasts.

In wound tissue, JY < 10 kDa increased PTCH1, GLI1, and Cyclin D1 protein expression in a concentration‐dependent manner (Figure 5A,B). Similar changes were observed in L929 cells treated with JY < 10 kDa (Figure 5C,D). GLI1 is a widely used transcriptional readout of Hh pathway activity, while Cyclin D1 is a downstream cell‐cycle regulator that may connect Hh activation with fibroblast proliferation. PTCH1 is a negative‐feedback receptor whose transcription is also induced by Hh signaling. Therefore, the simultaneous elevation of GLI1, PTCH1, and Cyclin D1 is consistent with activation of Hh pathway‐associated transcriptional responses and provides a plausible molecular link between JY < 10 kDa treatment and the observed increases in fibroblast proliferation and activation.

**FIGURE 5:**
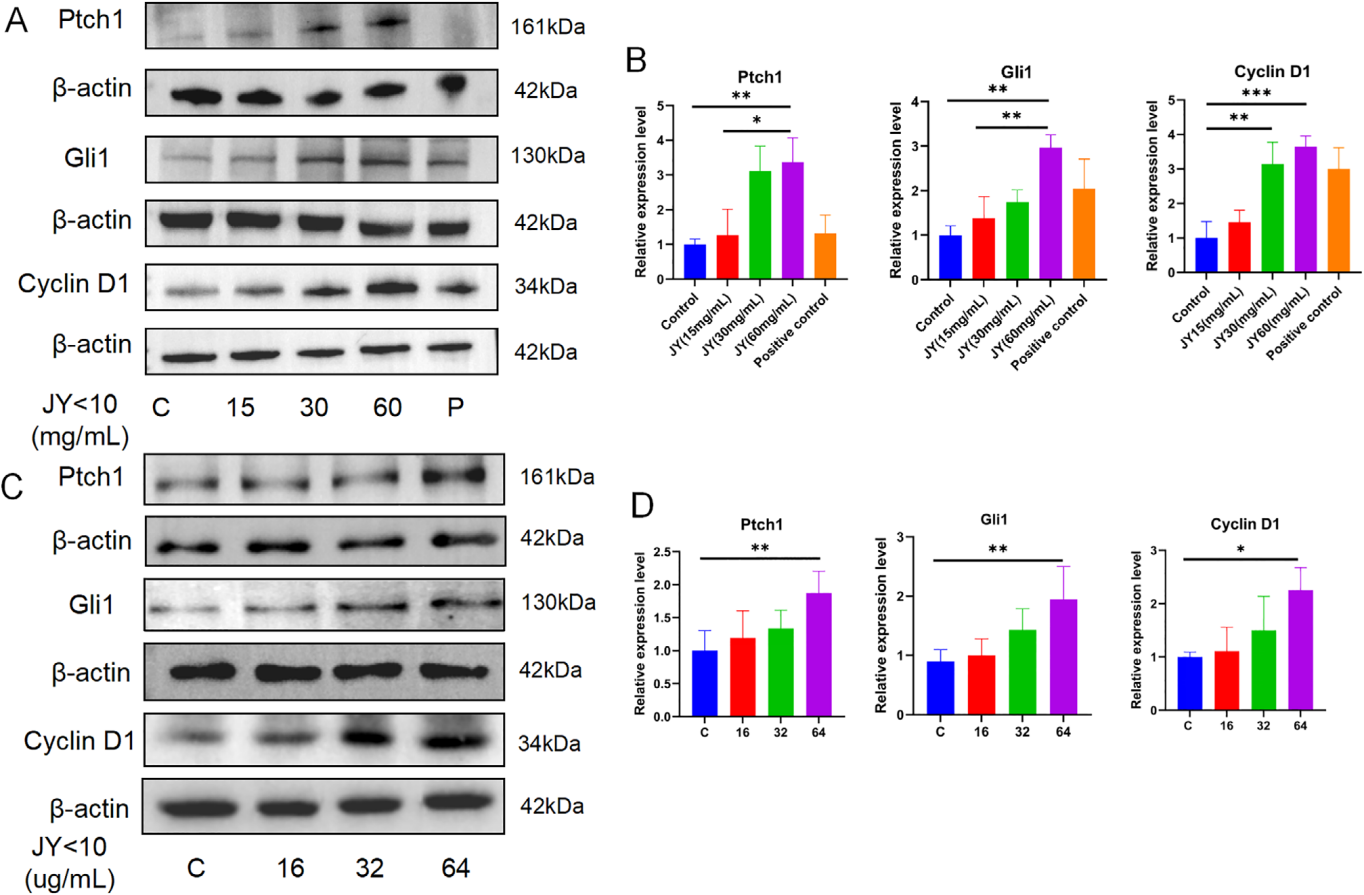
JY < 10 kDa activates the Hh signaling pathway (A): Expression of Hh signaling pathway marker proteins was examined in full‐thickness skin wounds of mice treated with different concentrations of JY < 10 KDa. (B):The optical density of all Western blot bands was analyzed and normalized, *n* = 3, *p < 0.05, **p < 0.01, ***p < 0.001. (C): Expression of Hh signaling pathway marker proteins was assessed in L929 fibroblasts following treatment with different concentrations of JY < 10 KDa. (D):The optical density of all Western blot bands was analyzed and normalized, n = 3, *p < 0.05, **p < 0.01, *** *p* < 0.001.

Canonical Hh signaling is initiated when an Hh ligand relieves PTCH1‐mediated inhibition of SMO, allowing SMO‐dependent signaling through the primary cilium and subsequent regulation of GLI transcription factors. The present experiments did not directly measure Hh ligand levels, SMO localization, GLI1 transcriptional activity, or primary‐cilium events. This will be further explored in our future research.

### 3.6 JY < 10 kDa Promotes Fibroblast Activation via the Hh Pathway

To determine whether Hh signaling is functionally required for the effects of JY < 10 kDa, L929 cells were treated with JY < 10 kDa at 64 µg/mL alone or in combination with the SMO inhibitor vismodegib at 10 µM. JY < 10 kDa significantly increased EdU incorporation, whereas co‐treatment with vismodegib markedly reduced this proliferative response (Figure 6A). Similarly, the enhanced scratch closure and transwell migration induced by JY < 10 kDa were substantially attenuated by Hh pathway inhibition (Figure 6B,C). The molecular findings were consistent with the functional assays. JY<10 kDa increased PCNA, α‐SMA, and type I collagen expression, whereas vismodegib co‐treatment suppressed these changes (Figure 6D,E). These rescue experiments indicate that Hh signaling is not merely correlated with JY < 10 kDa treatment but contributes functionally to the regulation of fibroblast proliferation, migration, activation, and matrix production. Taken together, the data support the following working model: the JY < 10 kDa fraction activates Hh pathway‐associated signaling in fibroblasts, leading to increased GLI1‐related transcriptional activity and induction of cell‐cycle and repair‐associated proteins such as Cyclin D1, PCNA, α‐SMA, and collagen. The resulting enhancement of fibroblast proliferation, migration, myofibroblast‐like activation, and ECM production contributes to accelerated wound closure and improved tissue regeneration (Figure 7).

**FIGURE 6:**
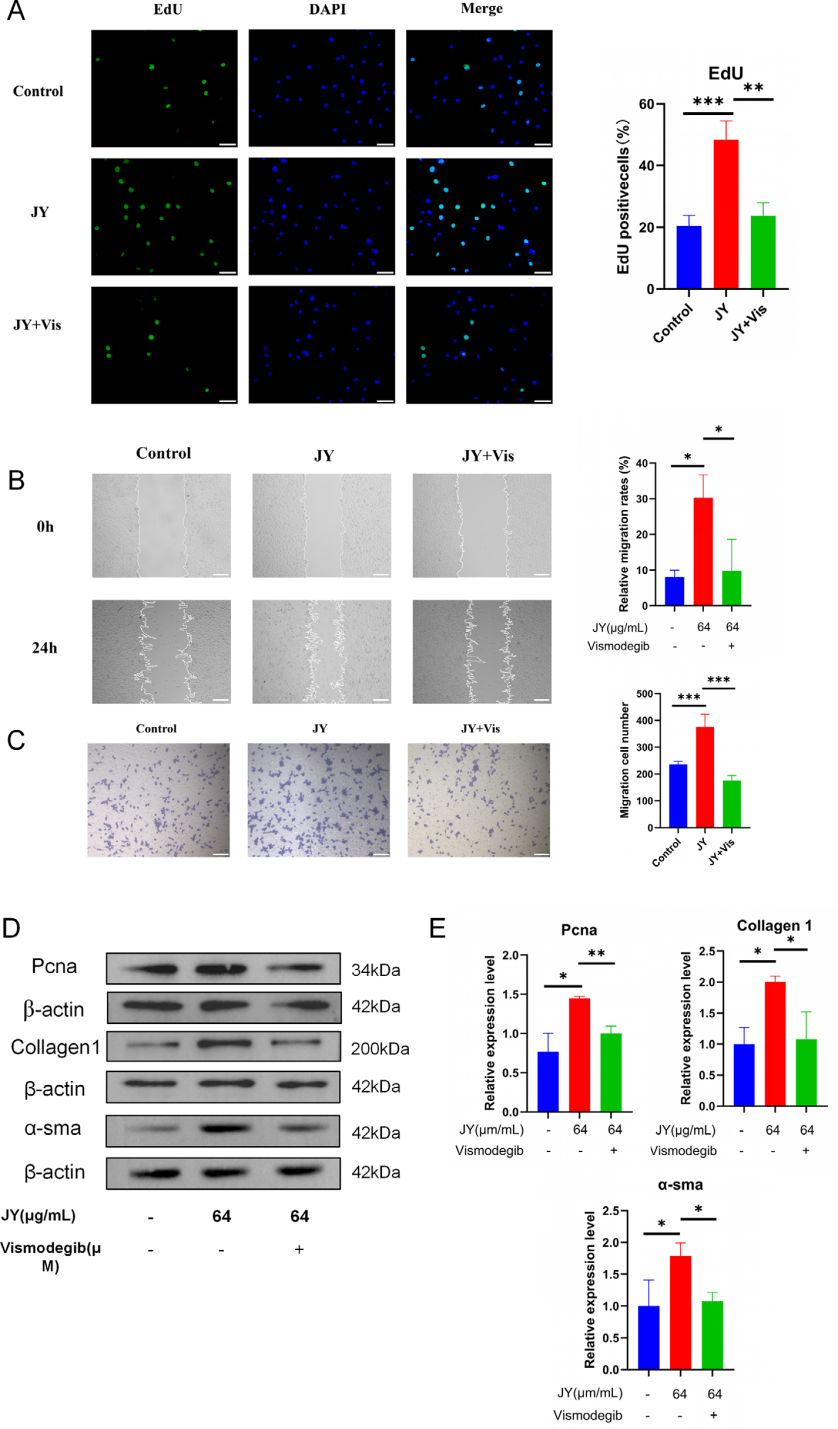
JY < 10 kDa promotes fibroblast activation via the Hh signaling pathway. (A): Quantitative analysis of cell proliferation by EdU assay following vismodegib treatment. Data are presented as the mean ± standard deviation (SD) from three independent experiments. *p < 0.05, **p < 0.01, ***p < 0.001, (Scale bar: 100 µm). (B) A wound scratch assay was performed following vismodegib treatment. Representative micrographs of the wound edges at 0 and 24 h post‐treatment with either JY or vismodegib are shown. The migration rate was quantified and is presented as the percentage of initial wound closure, *n* = 3, (Scale bar: 100 µm). (C) Cell migration was evaluated using a transwell assay following treatment with JY or vismodegib. Representative images of migrated cells (Scale bar: 100 µm) and the corresponding quantitative analysis are shown. Data are presented as the Mean ± SD, *n* = 5, *p < 0.05, **p < 0.01, ***p < 0.001. (D) Expression of cell proliferation and activation marker proteins was assessed following treatment with the Hh pathway inhibitor vismodegib. (E) The optical density of all Western blot bands was analyzed and normalized. *n* = 3, *p < 0.05, **p < 0.01, ***p < 0.001.

**FIGURE 7:**
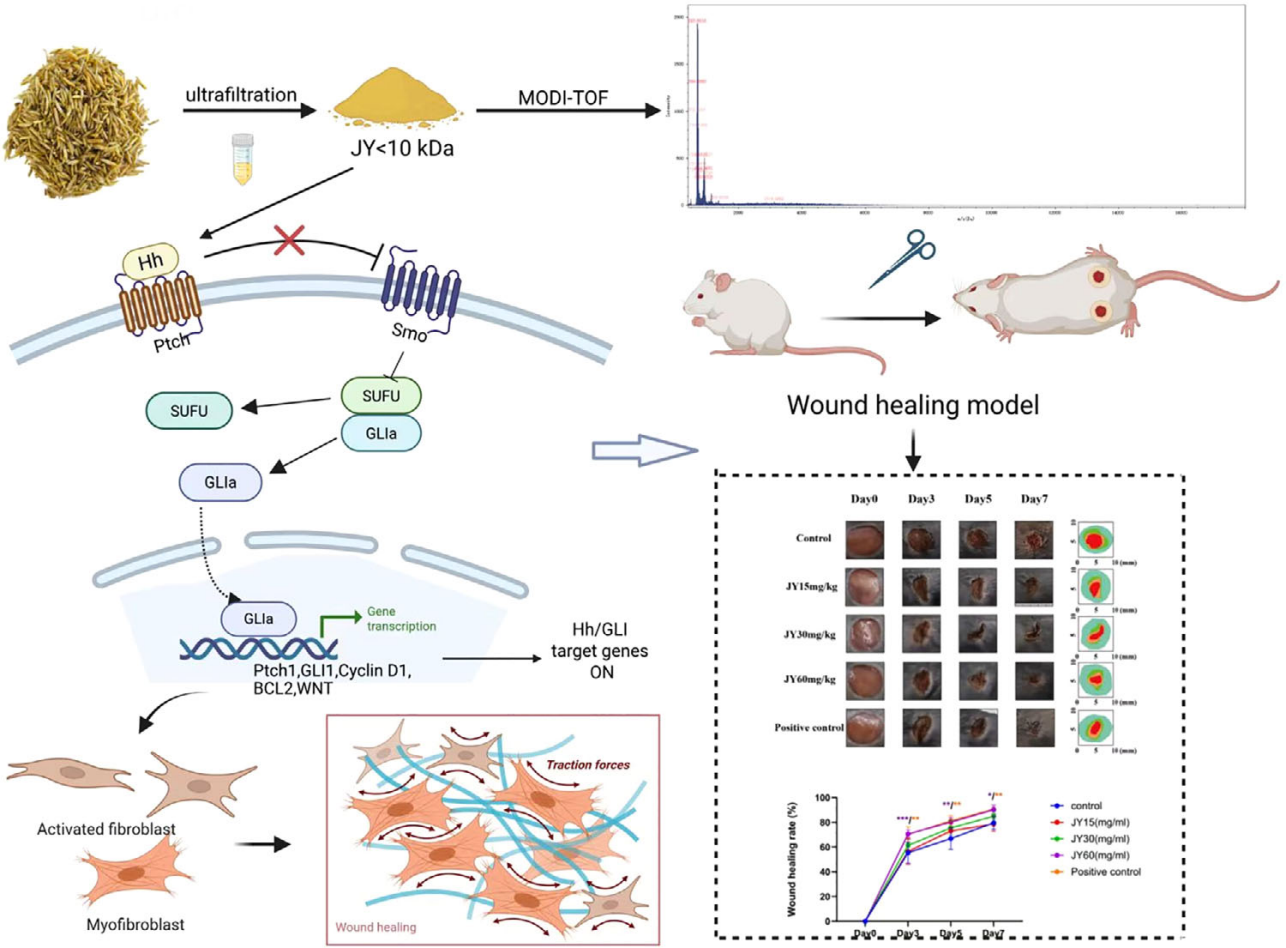
JY < 10 kDa accelerates wound healing via Hh signaling, which enhances fibroblast proliferation, migration, myofibroblast activation, and ECM production.

## 4 Conclusions

In a mouse full‐thickness skin wound model, JY < 10 kDa significantly accelerated wound closure, reduced inflammatory cell infiltration, promoted granulation tissue formation, and enhanced collagen deposition, demonstrating considerable therapeutic potential. In vivo and in vitro analyses verified that JY < 10 kDa upregulates the expression of α‐SMA, PCNA, and collagen, thereby enhancing fibroblast proliferation and activation and accelerating acute wound healing.

Further investigation demonstrated that JY < 10 kDa activates the Hh signaling pathway, which regulates fibroblast proliferation, activation, and collagen production. Rescue experiments using a Hh pathway inhibitor [34] confirmed that this pathway is essential for the observed effects, establishing it as a key mechanism underlying JY < 10 kDa‐induced wound healing.

In summary, this study elucidates that JY < 10 kDa promotes full‐thickness skin wound healing by activating the Hh signaling pathway, which in turn enhances fibroblast proliferation, activation, and collagen synthesis. Our findings systematically clarify the Hh‐fibroblast axis as a central mechanistic pathway and provide a novel strategic foundation for developing natural product‐based therapeutics for wound management.

## 5 Limitations

This study has several limitations. First, JY < 10 kDa is a heterogeneous low‐molecular‐weight fraction, and its precise chemical composition, peptide sequences, and active constituents remain unidentified. Further bioactivity‐guided fractionation and LC‐MS/MS analysis are required. Second, the experiments used a small number of healthy male mice with acute wounds and a short observation period; therefore, the findings cannot yet be extrapolated to chronic diabetic, ischemic, infected, or otherwise refractory wounds. Third, the study primarily examined L929 fibroblasts and did not determine whether JY < 10 kDa selectively affects fibroblasts or also regulates keratinocytes [38], endothelial cells [39, 40], macrophages [41, 42], or other wound‐related cells. Fourth, the increased expression of PTCH1, GLI1, and Cyclin D1, together with the inhibitory effect of vismodegib, supports Hh pathway involvement but does not establish direct or exclusive pathway activation. Additional genetic and direct pathway assays are needed. Finally, the dosing parameters, formulation reproducibility, long‐term safety, pharmacokinetics, and effects on scar remodeling and tissue function require further evaluation. Thus, JY < 10 kDa should currently be considered a promising preliminary lead rather than a fully characterized therapeutic candidate.

## Author Contributions

**Kun Li**: funding acquisition, writing – review and editing, writing – original draft. **Yunpu Sun**: writing – original draft, validation. **Lijie Pang** and **Tiantian Gao**: software, methodology, resources. **Yunpeng Diao**: writing – original draft. **Shuyuan Fan**: writing – original draft.

## Conflicts of Interest

The authors declare no conflicts of interest.

## Supporting information




**Supporting File 1**: cbdv71671‐sup‐0001‐SuppMat.docx

## Data Availability

Data will be made available on request

## References

[1] Y. Zhao , X. Wang , R. Qi , et al., “Recent Advances of Natural‐Polymer‐Based Hydrogels for Wound Antibacterial Therapeutics,” Polymers 15, no. 15 (2023): 3305, 10.3390/polym15153305. PMC10422483 37571202

[2] L. M. Morton and T. J. Phillips , “Wound Healing and Treating Wounds: Differential Diagnosis and Evaluation of Chronic Wounds,” Journal of the American Academy of Dermatology 74, no. 4 (2016): 589–605, 10.1016/j.jaad.2015.08.068, quiz ‐6. 26979352

[3] Y. Peng , Y. Wu , Z. Fu , et al., “Amphibian‐Derived Peptide OA‐AL14 Alleviates UVB‐Induced Skin Photoaging via Autophagy Activation Mediated by the AMPK/mTOR Pathway,” Integrative Zoology (forthcoming), 10.1111/1749-4877.70134. 42241584

[4] M. Rodrigues , N. Kosaric , C. A. Bonham , et al., “Wound Healing: A Cellular Perspective,” Physiological Reviews 99, no. 1 (2019): 665–706, 10.1152/physrev.00067.2017. PMC6442927 30475656

[5] D. Correa‐Gallegos , D. Jiang , and Y. Rinkevich , “Fibroblasts as Confederates of the Immune System,” Immunological Reviews 302, no. 1 (2021): 147–162, 10.1111/imr.12972. 34036608

[6] B. Hinz , “The Role of Myofibroblasts in Wound Healing,” Current Research in Translational Medicine 64, no. 4 (2016): 171–177, 10.1016/j.retram.2016.09.003. 27939455

[7] A. Abdullahi , S. Amini‐Nik , and M. G. Jeschke , “Animal Models in Burn Research,” Cellular and Molecular Life Sciences 71, no. 17 (2014): 3241–3255, 10.1007/s00018-014-1612-5. PMC4134422 24714880

[8] P. Pakshir , N. Noskovicova , M. Lodyga , et al., “The Myofibroblast at a Glance,” Journal of Cell Science 133, no. 13 (2020): jcs227900, 10.1242/jcs.227900. 32651236

[9] P. Monika , P. V. Waiker , M. N. Chandraprabha , et al., “Myofibroblast Progeny in Wound Biology and Wound Healing Studies,” Wound Repair and Regeneration 29, no. 4 (2021): 531–547, 10.1111/wrr.12937. 34009713

[10] J. Du , X. Liu , C. W. Y. Wong , et al., “Direct Cellular Reprogramming and Transdifferentiation of Fibroblasts on Wound Healing‐Fantasy or Reality?,” Chronic Diseases and Translational Medicine no. 3 (2023): 191–199, 10.1002/cdt3.77. PMC10497843 37711868

[11] B. Hinz and D. Lagares , “Evasion of Apoptosis by Myofibroblasts: A Hallmark of Fibrotic Diseases,” Nature Reviews Rheumatology 16, no. 1 (2020): 11–31, 10.1038/s41584-019-0324-5. PMC7913072 31792399

[12] P. Martin and R. Nunan , “Cellular and Molecular Mechanisms of Repair in Acute and Chronic Wound Healing,” British Journal of Dermatology 173, no. 2 (2015): 370–378, 10.1111/bjd.13954. PMC4671308 26175283

[13] G. Broughton , J. E. Janis , and C. E. Attinger , “Wound Healing: An Overview,” Plastic and Reconstructive Surgery 117, no. 7 Suppl (2006): 1e–S–32e–S, 10.1097/01.prs.0000222562.60260.f9. 16801750

[14] G. S. Schultz , R. G. Sibbald , V. Falanga , et al., “Wound Bed Preparation: A Systematic Approach to Wound Management,” Wound Repair and Regeneration 11, no. Suppl 1 (2003): S1–28, 10.1046/j.1524-475x.11.s2.1.x. 12654015

[15] N. C. Schaper , J. J. van Netten , J. Apelqvist , et al., “Practical Guidelines on the Prevention and Management of Diabetes‐Related Foot Disease (IWGDF 2023 update),” Diabetes Metabolism Research and Reviews 40, no. 3 (2024): e3657, 10.1002/dmrr.3657. 37243927

[16] J. Kim , O. Nomkhondorj , C. Y. An , et al., “Management of Diabetic Foot Ulcers: A Narrative Review,” Journal of Yeungnam Medical Science 40, no. 4 (2023): 335–342, 10.12701/jyms.2023.00682. PMC10626295 37735855

[17] L. Su , Y. Jia , L. Fu , et al., “The Emerging Progress on Wound Dressings and Their Application in Clinic Wound Management,” Heliyon 9, no. 12 (2023): e22520, 10.1016/j.heliyon.2023.e22520. PMC10709065 38076148

[18] C. Nüsslein‐Volhard and E. Wieschaus , “Mutations Affecting Segment Number and Polarity in Drosophila,” Nature 287, no. 5785 (1980): 795–801, 10.1038/287795a0. 6776413

[19] J. E. Ming , E. Roessler , and M. Muenke , “Human Developmental Disorders and the Sonic Hedgehog Pathway,” Molecular Medicine Today 4, no. 8 (1998): 343–349, 10.1016/s1357-4310(98)01299-4. 9755453

[20] C. Sánchez‐Camacho , J. Rodríguez , J. M. Ruiz , et al., “Morphogens as Growth Cone Signalling Molecules,” Brain Research Reviews 49, no. 2 (2005): 242–252, 10.1016/j.brainresrev.2004.10.004. 16111553

[21] S. Frech , A. Forsthuber , A. Korosec , et al., “Hedgehog Signaling in Papillary Fibroblasts is Essential for Hair Follicle Regeneration During Wound Healing,” Journal of Investigative Dermatology 142, no. 6 (2022): 1737–1748.e5, 10.1016/j.jid.2021.11.026. 34922948

[22] L. P. Lai and J. Mitchell , “Indian Hedgehog: Its Roles and Regulation in Endochondral Bone Development,” Journal of Cellular Biochemistry 96, no. 6 (2005): 1163–1173, 10.1002/jcb.20635. 16187314

[23] A. M. Clark , K. K. Garland , and L. D. Russell , “Desert Hedgehog (Dhh) Gene is Required in the Mouse Testis for Formation of Adult‐Type Leydig Cells and Normal Development of Peritubular Cells and Seminiferous Tubules,” Biology of Reproduction 63, no. 6 (2000): 1825–1838, 10.1095/biolreprod63.6.1825. 11090455

[24] M. Athar , X. Tang , J. L. Lee , et al., “Hedgehog Signalling in Skin Development and Cancer,” Experimental Dermatology 15, no. 9 (2006): 667–677, 10.1111/j.1600-0625.2006.00473.x. 16881963

[25] Y. Hu , L. Peng , X. Zhuo , et al., “Hedgehog Signaling Pathway in Fibrosis and Targeted Therapies,” Biomolecules 14, no. 12 (2024): 1485, 10.3390/biom14121485. PMC11727624 39766192

[26] X. Liu , J. Liu , B. Li , et al., “First Identification of an Amphibian‐Derived Peptide With Anti‐Vascular Aging Activity,” Zoological Research 47, no. 3 (2026): 748–764, 10.24272/j.issn.2095-8137.2025.499. PMC13647062 42267563

[27] Z. Q. Ru , Y. T. Wu , C. Y. Yang , et al., “Ultra‐short Cyclic Peptide Cy (RL‐QN15) Acts as a TLR4 Antagonist to Expedite Oral Ulcer Healing,” Zoological Research 46, no. 5 (2025): 1187–1202, 10.24272/j.issn.2095-8137.2025.211. PMC12780494 41017403

[28] J. Wang , Y. Yang , Z. Ru , et al., “Food‐Derived Peptide RDP3 Mitigates Pyroptosis to Enhance Oral Mucosal Repair via the IL‐2Rβ/PI3K Axis,” NPJ Regenerative Medicine 11, no. 1 (2026): 40, 10.1038/s41536-026-00487-6. PMC13642947 42323320

[29] J. Zhou , R. Cha , Z. Wu , et al., “An Injectable, Natural Peptide Hydrogel With Potent Antimicrobial Activity and Excellent Wound Healing‐Promoting Effects,” Nano Today 49 (2023): 101801, 10.1016/j.nantod.2023.101801.

[30] Y. Li , Q. Jia , N. Liu , et al., “Peptide RL‐QN15 Regulates Functions of Epidermal Stem Cells to Accelerate Skin Wound Regeneration via the FZD8/β‐Catenin Axis,” Exploration 6, no. 3 (2026): 20240090, 10.1002/exp.20240090. PMC13317571 42382691

[31] C. J. Deng , Y. Wang , and X. W. Yang , “Amphibian‐derived Peptides as Novel Therapeutics for Skin Wound Healing: Mechanisms, Applications, and Challenges,” Zoological Research 47, no. 3 (2026): 727–747, 10.24272/j.issn.2095-8137.2026.076. PMC13647057 42267562

[32] T. Y. Wang , W. Wang , F. F. Li , et al., “Maggot Excretions/Secretions Promote Diabetic Wound Angiogenesis via miR18a/19a—TSP‐1 Axis,” Diabetes Research and Clinical Practice 165 (2020): 108140, 10.1016/j.diabres.2020.108140. 32277954

[33] F. Peng , J. Zong , T. Zhao , et al., “Anti‐Inflammatory and Immunomodulatory Effects of Polysaccharide Extracted From Wuguchong (Maggot) on 2,4‐dinitrochlorobenzene‐Induced Atopic Dermatitis in Mice,” Frontiers in Pharmacology 14 (2023): 1119103, 10.3389/fphar.2023.1119103. PMC10073476 37033625

[34] Z. Zhang , J. Yang , R. Liu , et al., “Inhibiting HMGCR Represses Stemness and Metastasis of Hepatocellular Carcinoma via Hedgehog Signaling,” Genes & Diseases 11, no. 5 (2024): 101285, 10.1016/j.gendis.2024.101285. PMC11252768 39022130

[35] R. E. Mirza , M. M. Fang , E. M. Weinheimer‐Haus , et al., “Sustained Inflammasome Activity in Macrophages Impairs Wound Healing in Type 2 Diabetic Humans and Mice,” Diabetes 63, no. 3 (2014): 1103–1114, 10.2337/db13-0927. PMC3931398 24194505

[36] L. Chambers , S. Woodrow , A. P. Brown , et al., “Degradation of Extracellular Matrix Components by Defined Proteinases From the Greenbottle Larva Lucilia Sericata Used for the Clinical Debridement of Non‐Healing Wounds,” British Journal of Dermatology 148, no. 1 (2003): 14–23, 10.1046/j.1365-2133.2003.04935.x. 12534589

[37] Y. Cai , K. Chen , C. Liu , et al., “Harnessing Strategies for Enhancing Diabetic Wound Healing From the Perspective of Spatial Inflammation Patterns,” Bioactive Materials 28 (2023): 243–254, 10.1016/j.bioactmat.2023.04.019. PMC10245071 37292231

[38] M. Zhao , B. Song , J. Pu , et al., “Electrical Signals Control Wound Healing Through Phosphatidylinositol‐3‐OH Kinase‐Gamma and PTEN,” Nature 442, no. 7101 (2006): 457–460, 10.1038/nature04925. 16871217

[39] Y. Liu , S. R. Cox , T. Morita , et al., “Hypoxia Regulates Vascular Endothelial Growth Factor Gene Expression in Endothelial Cells. Identification of a 5' enhancer,” Circulation Research 77, no. 3 (1995): 638–643, 10.1161/01.res.77.3.638. 7641334

[40] A. Cucina , V. Borrelli , B. Randone , et al., “Vascular Endothelial Growth Factor Increases the Migration and Proliferation of Smooth Muscle Cells Through the Mediation of Growth Factors Released by Endothelial Cells,” Journal of Surgical Research 109, no. 1 (2003): 16–23, 10.1016/s0022-4804(02)00042-2. 12591230

[41] C. D. Mills , “M1 and M2 Macrophages: Oracles of Health and Disease,” Critical Reviews in Immunology 32, no. 6 (2012): 463–488, 10.1615/critrevimmunol.v32.i6.10. 23428224

[42] F. O. Martinez and S. Gordon , “The M1 and M2 Paradigm of Macrophage Activation: Time for Reassessment,” F1000Prime Reports 6 (2014): 13, 10.12703/p6-13. PMC3944738 24669294

