# Supplementary material for: Wuguchong Bioactive Peptides Promote Wound Healing via Hedgehog Pathway‑Mediated Fibroblast Modulation
Source: Chem Biodivers. 2026 Oct 21;23(10):e71671. doi: 10.1002/cbdv.71671 (PMC13685478; doi:10.1002/cbdv.71671)
Supplement: Supplementary file 1 [file CBDV-23-e71671-s001.docx]

Supplementary figure.1 (A) Molecular Weight Distribution of <10 kDa Bioactive Components in JY by MALDI-TOF MS.

Supplementary figure.2 Statistics of inflammatory infiltrating cells in HE staining among different groups.  n = 3, *p < 0.05.

Supplementary figure.3 Quantitative analysis of the collagen positive area in different groups. n = 3, *p<0.05, **p<0.01, ***p<0.001.

Supplementary figure.4 IHC analysis for the expression of Collagen I of wound tissues from different groups. n = 3, *p<0.05，**p<0.01.

Supplementary figure.5 IHC analysis for the expression of α-SMA of wound tissues from different groups. n = 3, *p < 0.05.
